# Kinematic profiles suggest differential control processes involved in bilateral in-phase and anti-phase movements

**DOI:** 10.1038/s41598-019-40295-1

**Published:** 2019-03-01

**Authors:** Pei-Cheng Shih, Christopher J. Steele, Vadim Nikulin, Arno Villringer, Bernhard Sehm

**Affiliations:** 1 Department of Neurology, Max Planck Institute for Human Cognitive and Brain Sciences, Leipzig, Germany; 2 Department of Psychology, Concordia University, Montreal, Quebec, Canada; 3 Department of Cognitive Neurology, University of Leipzig, Leipzig, Germany

## Abstract

In-phase and anti-phase movements represent two basic coordination modes with different characteristics: during in-phase movements, bilateral homologous muscle groups contract synchronously, whereas during anti-phase movements, they contract in an alternating fashion. Previous studies suggested that in-phase movements represent a more stable and preferential bilateral movement template in humans. The current experiment aims at confirming and extending this notion by introducing new empirical measures of spatiotemporal dynamics during performance of a bilateral circle drawing task in an augmented-reality environment. First, we found that anti-phase compared to in-phase movements were performed with higher radial variability, a result that was mainly driven by the non-dominant hand. Second, the coupling of both limbs was higher during in-phase movements, corroborated by a lower inter-limb phase difference and higher inter-limb synchronization. Importantly, the movement acceleration profile between bilateral hands followed an in-phase relationship during in-phase movements, while no specific relationship was found in anti-phase condition. These spatiotemporal relationships between hands support the hypothesis that differential neural processes govern both bilateral coordination modes and suggest that both limbs are controlled more independently during anti-phase movements, while bilateral in-phase movements are elicited by a common neural generator.

## Introduction

Coordinating both upper limbs is essential for most daily tasks, such as typing, eating, using tools or playing an instrument. While for healthy young subjects, bilateral coordinated activities are usually easy and effortless to conduct, performance often declines in the elderly or patients with neurological diseases as a consequence of neuromuscular changes^1,2^. However, even in healthy young subjects, the effort to accurately perform bilateral activities varies between different coordination patterns^3^.

Out of the broad repertoire of possible bilateral patterns, two dominant coordination modes that require simultaneous movements of both limbs have been investigated in the past since they represent templates of two basic movement modes: in-phase movements and anti-phase movements^3,4^. During in-phase movements, both hands move in a mirror-symmetrical pattern with respect to the body midline, thus corresponding to simultaneously recruited bilateral homologous muscle groups, while during anti-phase movements, homologous muscle groups are activated in an alternating fashion. Starting from early seminal work by Kelso and colleagues^5^, a consistent finding throughout studies is, that in-phase movements may be performed more accurately and effortlessly (i.e., with less attentional load) as compared to anti-phase movements^6–9^. For example, in-phase movements can be performed accurately without practice, while anti-phase movements often require training to be performed accurately^9^. In this vein, both behavioral experiments and simulation studies have demonstrated that, in an anti-phase condition, the movement might unintentionally change to an in-phase movement as movement frequency is increased. The opposite phase transition (from an in-phase to an anti-phase movement), on the other hand, rarely naturally occurs^10,11^. Furthermore, it was shown that an increase in circle drawing movement frequency, especially in anti-phase movements, results in a decrease in accuracy (particularly evident in the non-dominant hand) or even a phase-reversal of the non-dominant hand^12,13^. Based on this, it may be argued that there is a guiding influence of the dominant hand over the non-dominant hand in bilateral coordination that is less pronounced during in-phase movements. Taken together, this evidence supports the general idea, that in-phase movements represent a more basic bilateral coordination mode of the human motor system than anti-phase movements.

Previous studies have quantified inter-limb performance during bilateral coordination using indices such as the mean and standard deviation of the phase difference between hands^12,14–16^. With these measures, it has been demonstrated that a larger average phase difference between hands (with the dominant hand leading), and higher variability in phase difference is present during anti-phase movements compared to in-phase^17^. However, these studies usually have low sample size between 8 to 16, which resulted in a lower statistical power. Considering the current reproducibility crisis in science^18,19^, the current experiment aimed at using not only a larger sample size to provide a better statistical power on the results but also utilized a more precise measurement tool. Here, we adapted the circle-drawing task into KINARM (BKIN Technologies Ltd, Ontario, Canada), a device that has a temporal resolution of 1000 Hz and spatial resolution in the millimeter range to assess upper-limb bilateral coordination. Taking advantage of the high temporal and spatial resolution of the device, we computed measurements that captured movement variabilities, inter-limb phase synchronization, as well as a metric quantifying the inter-limb acceleration relationship during different bilateral coordination patterns. Based on the previous literature, we hypothesized that both spatial and temporal measures will be differentially affected by in-phase movements and anti-phase movements; more specifically, (1) in-phase movement can be performed with lower spatial and temporal variability, and (2) the two hands will be spatially and temporally more synchronized with each other in the in-phase condition.

## Methods

### Participants

Thirty healthy young volunteers (age: 26.24 ± 3.13 years, 15 male) participated in this study. All participants were right-handed according to the Edinburgh Handedness Inventory^20^ (score: 88.52 ± 15.77). Participants did not have experience in the testing paradigm and were naive to the purpose of the study. The experiment was approved by the ethics committee of the University of Leipzig and performed in agreement with the Declaration of Helsinki; all participants gave written informed consent to join the experiment.

### Experimental device

**Figure 1:**
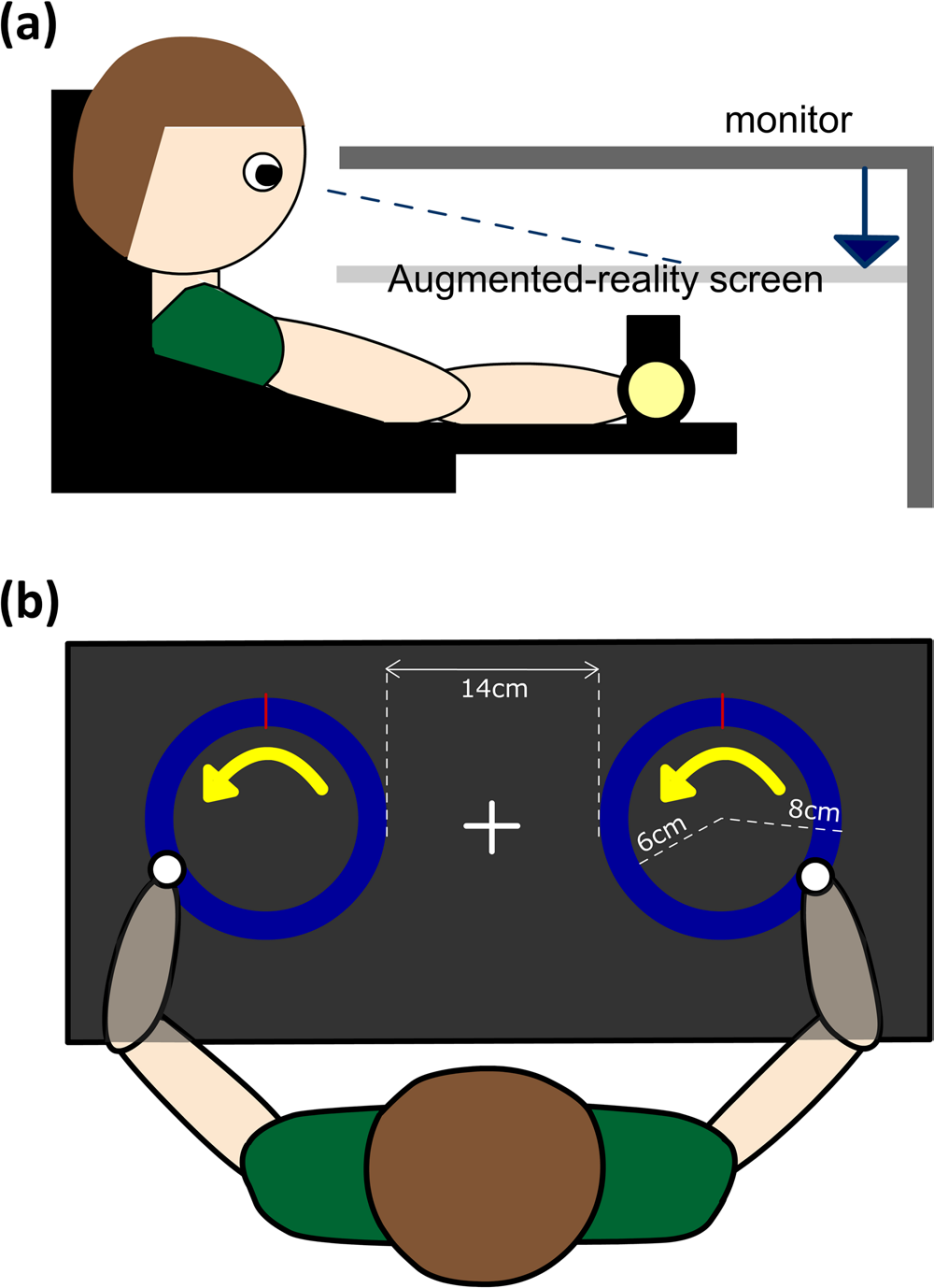
Experimental setup based on the KINARM upper limb exoskeleton device. **(a)** Participants put their arms on the gravity-support platforms, with the hands holding the handrails. The augmented-reality screen displayed the paradigm projected by the monitor. (**b**) The relative position of the participant and the circle position. The white fixation cross is presented at the midline of participants. The yellow arrows inside the circle path were used to indicate the required movement direction. The red bar indicates the starting hand position for the task.

The experiments were performed using the KINARM upper limb robotic exoskeleton system. KINARM has been widely used as a motor assessment device. It is capable of recording movements in the millimeter range at a sampling rate of 1000 Hz, which help to better quantify sensory and motor characteristics in healthy subjects as well as subjects with neurological disorders^21,22^. The KINARM device includes a height-adjustable chair with bilateral arm-gravitational-support platforms and handrails, a monitor linked to the operator’s computer and a screen under the monitor to present the task paradigm (Fig. 1a). This environment allows participants to perform two-dimensional planar shoulder and elbow movements under the presentation screen, which means both arm movements and the visual display of the motor task are within the same workspace. Participants’ movements were continuously recorded by the Dexterit-E (3.5 v, BKIN Technologies Ltd, Ontario, Canada) software during the task performance at a sampling rate of 1000 Hz. The recorded data were saved automatically to a c3d data file, containing the hand position coordinates (x,y) and the movement velocity along the transverse plane.

### Circle Drawing Task

We developed the circle drawing task on Simulink (R2015, The MathWorks, USA) and Dexterit-E to probe upper-limb coordination. As shown in Fig. 1b, two target circles were displayed side by side on the screen with the distance between their centers set at 22 cm. The inner/outer diameter of each circle is 6/8 cm, which creates a 2-cm-thick circle path (shown in blue). The distance and size of the circles were determined by pilot testing with young adults. A white fixation cross was positioned between the two circles. A red vertical line at the top of each circle indicated the starting point of the task, and a yellow arrow was projected inside the circle to point out the active hand(s) and the upcoming movement direction(s). An auditory metronome (0.85 Hz) started at the beginning of each trial in order to provide a cue for the required movement frequency. The frequency of the metronome was selected based on a pilot experiment and provided participants with a comfortable speed for rhythmic movements without potential phase transition.

**Figure 2:**
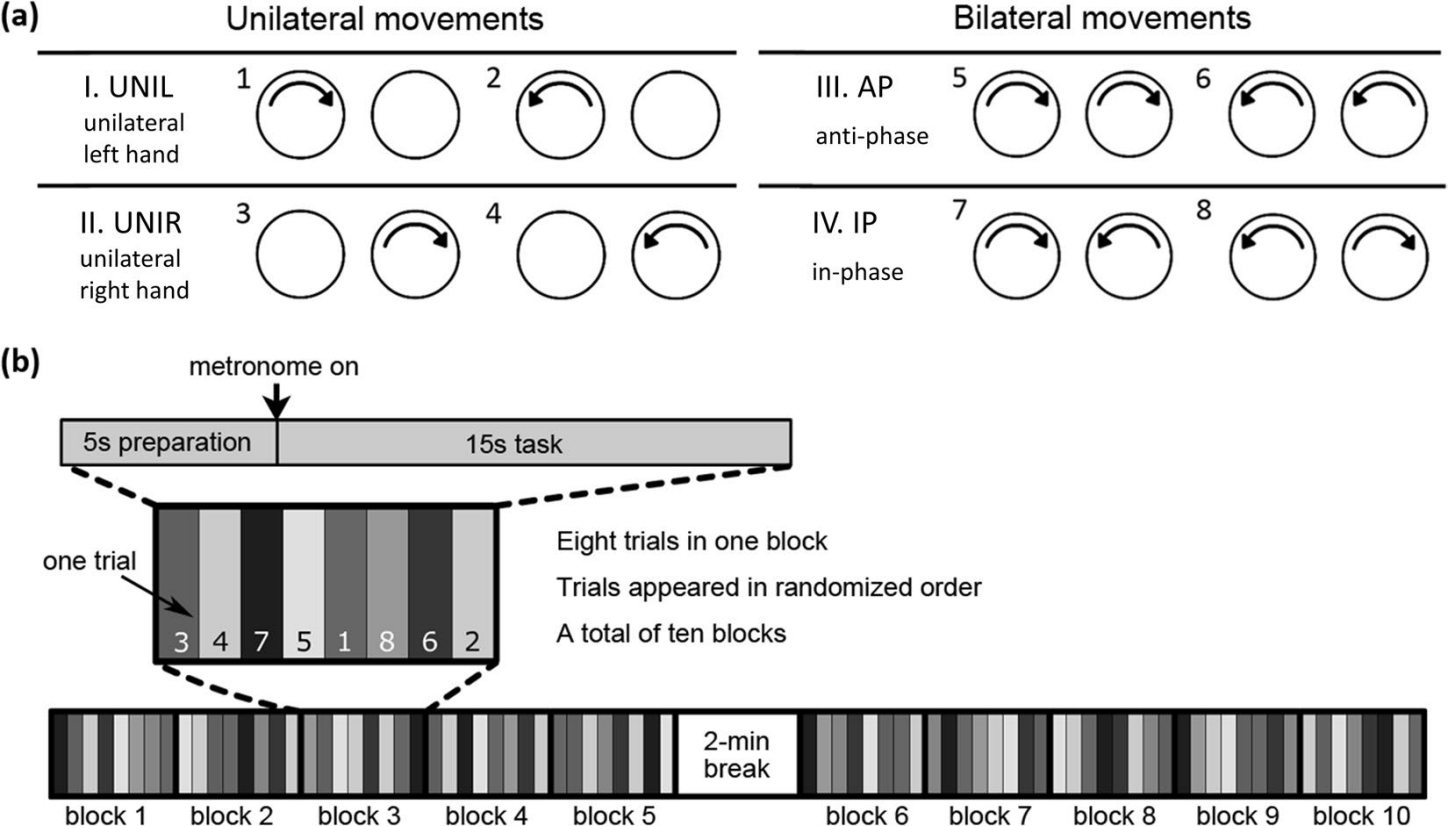
(**a**) Testing conditions. I. Unilateral left hand (UNIL). II. Unilateral right hand (UNIR). III. Anti-phase condition. IV. In-phase condition. **(b)** Task design. Eight trials (eight movement patterns) were displayed as a 15 s trial in randomized order within one block, and a total of 10 blocks were performed in the whole experiment. Before each of the 15 s trial started, participants had to hold their hands on the starting point for 5 seconds.

There were a total of eight testing conditions, which were classified into four main movement patterns (Fig. 2a): left unilateral movements (UNIL), right unilateral movements (UNIR), bilateral anti-phase and bilateral in-phase movements. As shown in Fig. 2b, each movement condition was conducted in a 15 s trial, preceded by a 5 s preparation phase. Participants were instructed to (1) check the upcoming movement direction(s) on the screen and then put the active hand(s) on the starting point(s); (2) wait for the start of the trial as indicated by the auditory metronome (which sounded 5 seconds after the hand(s) was/were at the starting point); (3) draw continuous circles in synchrony with the metronome, in a way that the hands are at the starting point during the sound of the metronome; (4) try to keep the hands within the circle path. Participants were instructed to focus their eyes on the white fixation cross during drawing to minimize head movement and attentional bias^14^.

Each condition was performed once in a randomized order in a block. There were a total of 10 blocks within the whole experiments, and a two minute break between blocks 5 and 6, resulting in the total time of approximately 30 minutes for the entire experiment. Before the experiment started, all participants had already practiced every movement condition once (in the order of condition 1–8, as shown in Fig. 2a) to be familiarized with circle size and metronome frequency. Hence, we did not observe a learning effect across performance of the experiment (Supplementary Material 1).

### Data processing and outcome measurements

All raw data files, containing hand position and velocity information, were imported into Matlab (R2017a, The MathWorks, USA) for offline processing using BKIN TOOLS and custom processing scripts. To specifically focus on the steady performance within one trial, we discarded the first two metronome cycles after the metronome started; thus, only the 3rd to 11th (inclusive) metronome cycles were analyzed. The 8 movement conditions (Fig. 2(a) 1–8) were pooled for analysis under the same category (i.e. unilateral left, unilateral right, in-phase and anti-phase; Fig. 2(a) I–IV), since the effect of movement direction on the behavioral indices was not of primary interest in this study. Please refer to Supplementary Material 2 for an analysis of the effects of movement direction on kinematic parameters. All indices in each condition were computed first on a single-trial basis and then averaged across the ten repeated trials of the same condition.

#### Intra-limb variability

We developed three measures to characterize spatiotemporal performance of each hand. In each trial, the center of mass of the circle trajectories was set at (0, 0) individually for each hand.

**Figure 3:**
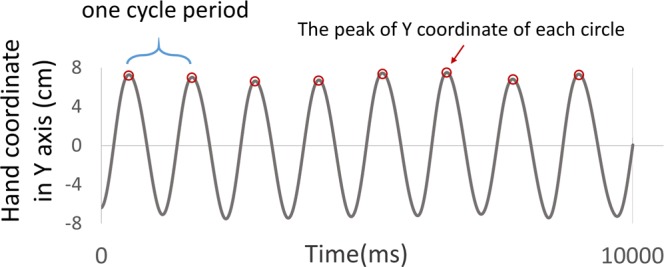
Assessment of mean cycle period and cycle period variability. An illustration of one representative participant’s trial. The red circles indicate the positions of the peak Y coordinate of each circle, and the peak-to-peak duration represents a cycle period (the blue window).

Mean cycle period and cycle period variability: We examined mean cycle period and cycle period variability to investigate participants’ ability to synchronize the movement with the metronome during the task. The cycle period was first estimated by computing the interval between the peak Y-coordinates in each trial (Fig. 3). Mean cycle period was the averaged cycle value within each trial. The cycle of the metronome was set at 1177 ms; therefore, a successfully synchronized performance should show a mean cycle period that is close to this value. Cycle period variability was defined as the coefficient of variation of the cycle periods within each trial. Lower cycle period variability indicates a more consistent ability to synchronize with the metronome within a trial.

Radial variability: this measurement is used to examine the spatial variability of movements within a trial^23^. The data was converted from Cartesian *(x, y)* to polar (*r, θ*) coordinates and the radius extracted from each sampling point. Within each trial, the coefficient of variation of the radius values across all sampling points was calculated to represent radial variability. A lower value indicates a more consistent trajectory during the drawing movement.

Peak speed variability: this measurement assesses the temporal variability of the repetitive circle drawing^9^, thus providing information on temporal consistency during the continuous movement. The peak velocity of each cycle was computed, and then the coefficient of variation across cycles was calculated to represent temporal variability. A lower value indicates that participants drew the 12 circles within a trial in a more consistent speed.

For all three indices, a lower value implies a more consistent spatial or temporal performance, while a higher value represents more variance in performance.

#### Inter-limb coupling measurements

**Figure 4:**
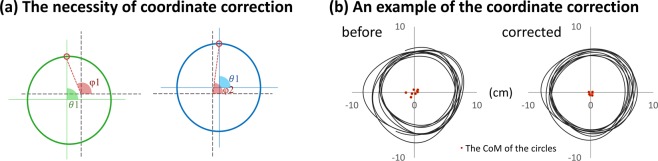
Coordinate correction. **(a)** The necessity of coordinate correction before calculating the phase values. From the Center of Mass (CoM) of the left (green) and right (blue) circles, the two small red dots both lie on 90 degrees of the circles (*θ*1 = *θ*2). However, when the two circles are lying on a common coordinate (black coordinate) with a spatial shift, the phase value of the two dots become different ($\phi 1\ne \phi 2$). **(b)** An example of coordinate correction. The participant had slight movement of the CoM between each cycle. After correction, the CoM between cycles becomes more stable. The tiny red dots represent the CoM of each circle.

We developed additional indices to examine the phase relationships between both limbs to assess how they interact with each other during the bilateral conditions. As a first step, we performed a curvature correction to reduce the effect of participants’ unintentional center-shifting on phase calculations. This was performed to avoid inaccuracy of the phase value based on center shifts (see Fig. 4a for a graphical explanation). We first estimated the centroid for each sampling point based on the circle cycle using least-squared fitting and then corrected its position^24^. This method preserved the phase relationship between each sampling point, while excluding the potential influence of spatial shifting on the phase calculation (Fig. 4b). As an additional information for participants’ task performance, the offset of the centroid is also reported (Supplementary Material 3). We found significant increases of centroid offset during the anti-phase condition in the left hand. Therefore, centroid correction is essential to reduce potential biases of the phase calculation from the spatial shift.

Three indices were then computed to measure inter-limb coordination ability in different bilateral conditions:

1. Mean phase difference between hands: we calculated the averaged phase difference value ${\phi }_{(R,L)}={\phi }_{R}(t)-{\phi }_{L}(t)$ in each trial to examine whether there is an effect of a particular hand leading. A positive value suggests that the right hand is in leading position, while a negative value indicates that the left hand is in leading position.
2. Phase synchronization index: we used phase synchronization index to quantify how well the two hands synchronized with each other. Since standard deviation is prone to errors in estimating circular variability as the data is periodic^25^, we adopted the phase synchronization index, which is instead based on the circular variance of the angular distribution, to prevent this problem^26^. It thus measures the angular deviation and quantifies how consistent the phase oscillation between the two hands are. The index is obtained by projecting the phase differences between two hands onto the unit circle and calculating the absolute value of the mean phase difference between hands:

1 $${synchronization}\,{index}=|\frac{1}{T}\sum _{t=1}^{T}{e}^{i[{\phi }_{R}(t)-{\phi }_{L}(t)]}|,$$

where ${\phi }_{R}(t)$ and ${\phi }_{L}(t)$ represents the unwrapped phase of the left and right hand during the sampling point *t*, and *T* represents the total amount of the sampling points in a trial. This index ranges from 0 to 1. A value close to zero indicates no phase synchronization, while 1 corresponds to perfect phase synchronization. Note that the mean phase difference itself does not affect the strength of the synchronization index. In addition, the result of the phase synchronization index was compared to the more “traditional” measure, standard deviation of the relative phase difference (see Supplementary Material 4).

Inter-limb acceleration index: this measure was used to examine whether the two hands are accelerating synchronously with each other. First, the speed data were smoothed using a third-order one-dimensional median filter through the Matlab medfilt1 function. Second, we took the differentiation of the angular speed to obtain the instantaneous rate of change of speed from the left hand (*aL*) and right hand (*aR*). Then, we calculated the Pearson correlation coefficient (Matlab corr function) from the respective acceleration values. This provides a bounded value that examines the tendency of bilateral hands’ acceleration relationship. Value −1 denotes a complete anti-phase acceleration relationship between hands; value +1 indicates a complete in-phase acceleration relationship between hands; while a value close to 0 stands for no specific phase relationship.

### Statistical analyses

All statistical analyses were performed using SPSS 20 (IBM, NY, USA), and results are presented as mean ± SD. For spatiotemporal variability, paired-t tests were used to compare the performance in the unilateral conditions between left and right hand (UNIL versus UNIR); two-way repeated-measures Analysis of Variance (ANOVA) were used in the bilateral conditions for comparing anti-phase movements and in-phase movements, which aimed at determining the effect of hand (left, right) and condition (anti-phase movements, in-phase movements). For inter-limb coupling measurements, we used paired-t tests to examine potential differences between anti-phase movements and in-phase conditions.

## Results

### Intra-limb performance

#### Mean cycle period and cycle period variability

We used mean cycle period and cycle period variability to investigate whether participants consistently synchronized with the metronome in all conditions.

**Figure 5:**
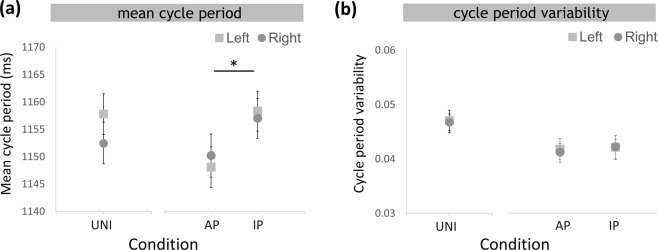
(**a**) Mean cycle period and **(b)** cycle period variability of the unilateral and the bilateral conditions. Values for UNI are depicted in grey, anti-phase movements in blue, in-phase movements in red. Values for left hand are shown as squares, right hand as circles.

In the unilateral conditions (Table 1), no significant difference between hands was found in both mean cycle period (t_(29)_ = 1.358, p = 0.185) and cycle period variability (t_(29)_ = 0.307, p = 0.761). For mean cycle period during bilateral conditions, there was no interaction between HAND and CONDITION (F_(1,29)_ = 2.196, p = 0.149, η^2^ = 0.070), no significant main effect of HAND (F_(1,29)_ = 0.208, p = 0.613, η^2^ = 0.075) but CONDITION (F_(1,29)_ = 5.028, p = 0.027, η^2^ = 0.410), suggesting that participants’ cycle period was closer to the optimal cycle (i.e. 1177 ms) during the in-phase compared to the anti-phase movements. For cycle period variability during bilateral conditions, there was no HAND × CONDITION interaction (F_(1,29)_ = 1.081, p = 0.205, η^2^ = 0.036), no significant main effect of HAND (F_(1,29)_ = 0.228, p = 0.636, η^2^ = 0.008) nor CONDITION (F_(1,29)_ = 0.365, p = 0.551, η^2^ = 0.012) (Fig. 5). Taken together, participants showed more accurate mean cycle period during the in-phase condition compared to the anti-phase condition, while no differences in cycle period variability were found.

**Table 1:** Intralimb parameters: mean cycle period, cycle period variability, radial variability and peak speed variability.

Condition	Left hand			Right hand		
Variable	UNIL	Anti-phase	In-phase	UNIR	Anti-phase	In-phase
Mean cycle period (ms)	1123.35 ± 32.14	1147.54 ± 20.72	1153.14 ± 20.80	1150.84 ± 21.69	1147.54 ± 20.72	1153.14 ± 20.80
Cycle period variability	0.0464 ± 0.0108	0.04205 ± 0.0015	0.0423 ± 0.0019	0.0457 ± 0.0109	0.0415 ± 0.0014	0.0042 ± 0.0021
Radial variability	0.156 ± 0.0029	0.158 ± 0.027	0.137 ± 0.026	0.136 ± 0.029	0.137 ± 0.028	0.135 ± 0.030
Peak speed variability	0.0374 ± 0.0013	0.0365 ± 0.0016	0.0376 ± 0.0011	0.0033 ± 0.0010	0.0034 ± 0.0012	0.0033 ± 0.0011

#### Radial variability

**Figure 6:**
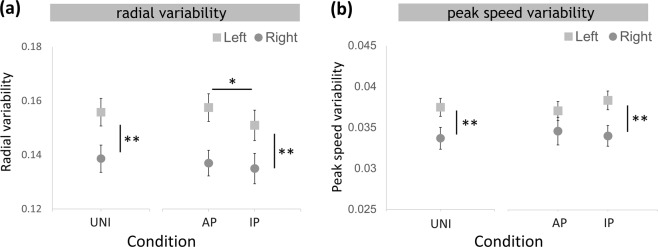
(**a**) Radial and (**b**) peak speed variability of the unilateral and the bilateral. *p < 0.05, **p < 0.001. Values for UNI are depicted in grey, anti-phase movements in blue, in-phase movements in red. Values for left hand are shown as squares, right hand as circles.

In the unilateral conditions, UNIL showed significantly higher (t_(29)_ = 7.564, p < 0.001) circle radial variability when compared with UNIR. In the bilateral conditions, anti-phase movements had greater radial variability compared to in-phase movements, and the left hand showed significant higher variability than the right hand (0.136 ± 0.05). These effects were supported by a main effect of HAND (F_(1,29)_ = 45.642, p < 0.001, η^2^ = 0.611) as well as CONDITION (F_(1,29)_ = 5.64, p = 0.022, η^2^ = 0.168). In addition, there was an interaction between HAND and CONDITION (F_(1,29)_ = 4.398, p = 0.045, η^2^ = 0.132, Fig. 6a) such that the non-dominant hand decreased in radial variability during in-phase movements, while performance of the dominant hand remained stable during anti-phase movements and in-phase movements.

#### Peak speed variability

In unilateral conditions, UNIL showed significant higher (t_(29)_ = 3.356, p = 0.002) peak speed variability than UNIR. In bilateral conditions, the left hand showed higher peak speed variability compared to the right hand, a result supported by a significant main effect of HAND (F_(1,29)_ = 20.410, p < 0.001, η^2^ = 0.413). There were no differences between anti-phase movements and in-phase movements (CONDITION: F_(1,29)_ = 0.132, p = 0.719, η^2^ = 0.005), nor was there an interaction HAND × CONDITION (F_(1,29)_ = 1.835, p = 0.187, η^2^ = 0.064, Fig. 6b).

Together, the results from the spatiotemporal analyses (i) confirmed that the non-dominant hand shows more variance than the dominant hand, and (ii) demonstrated that performance of the non-dominant hand is easier to be affected by movement modes; i.e., that radial variability is higher during the anti-phase movement mode.

### Inter-limb coupling measurements

#### Phase difference and phase synchronization index

**Figure 7:**
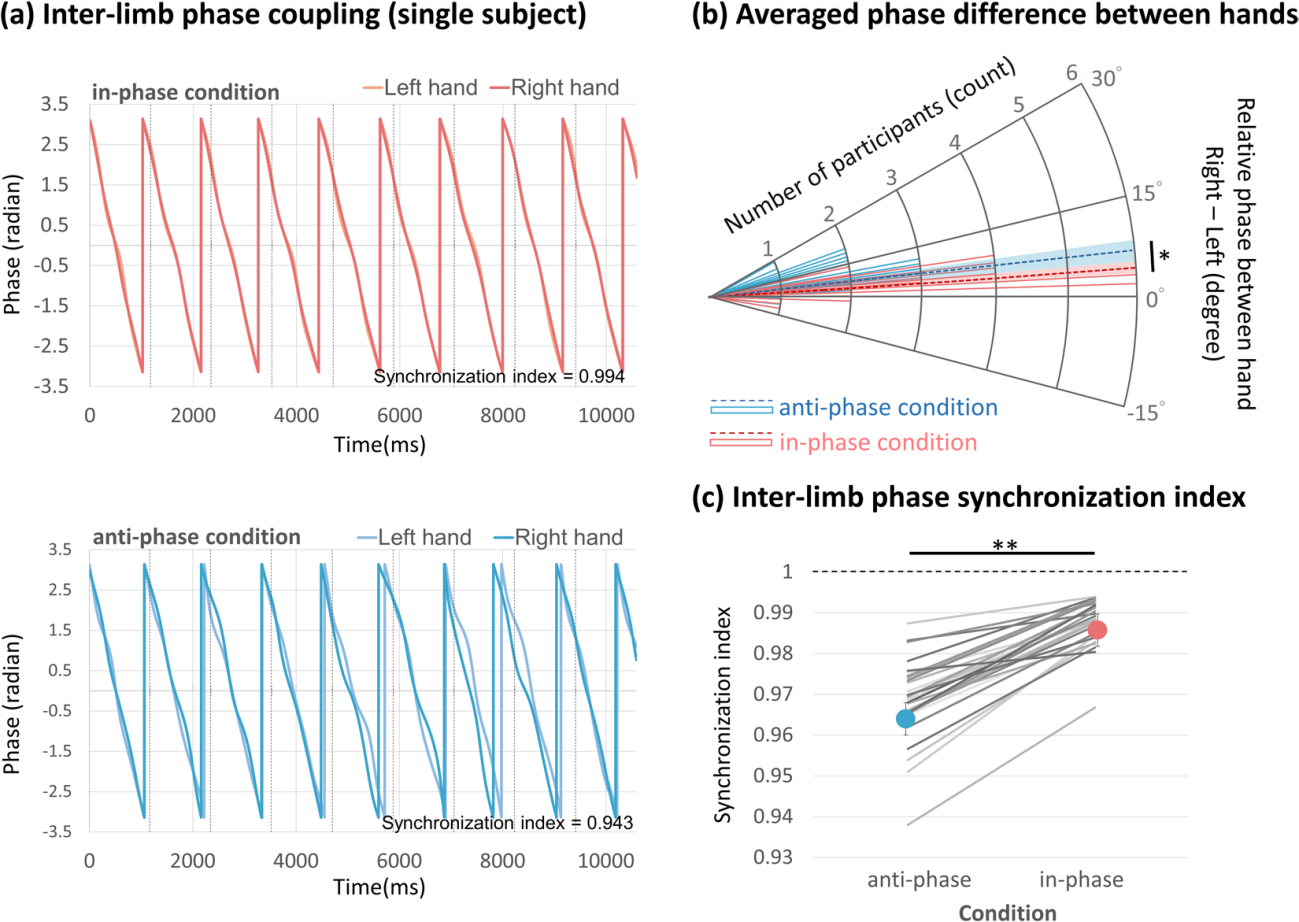
Inter-limb coordination indices. (**a**) An example of phase fluctuation within the trial. (**b**) Histogram of the phase difference during anti-phase movements and in-phase conditions (solid lines representing single subjects; dotted line ± shaded region representing mean ± SE). **(c)** Inter-limb phase synchronization index of anti-phase movements and in-phase condition. Grey lines illustrate the individual performance. *p < 0.05. **p < 0.001. Values for anti-phase movements in blue, in-phase movements in red. Values for each participants are shown as single grey lines.

For the mean phase difference, the averaged values during anti-phase movements (6.33 ± 1.204^0^) and in-phase movements (4.22 ± 0.628^0^) were both positive (indicating right-hand leading), and the paired-t test revealed that the phase difference between hands was significantly more pronounced (t_(29)_ = 2.777, p = 0.030) during anti-phase movements compared to in-phase movements (Fig. 7b). Although bilateral phase synchronization was consistently high in all conditions, we observed that participants performed the in-phase movements (0.98 ± 0.001) condition with greater (t_(29)_ = 8.276, p < 0.001) synchronization compared to anti-phase movements (0.96 ± 0.001) (see Fig. 7a for a single trial of an individual subject; Fig. 7c for group average).

#### Inter-limb acceleration index

**Figure 8:**
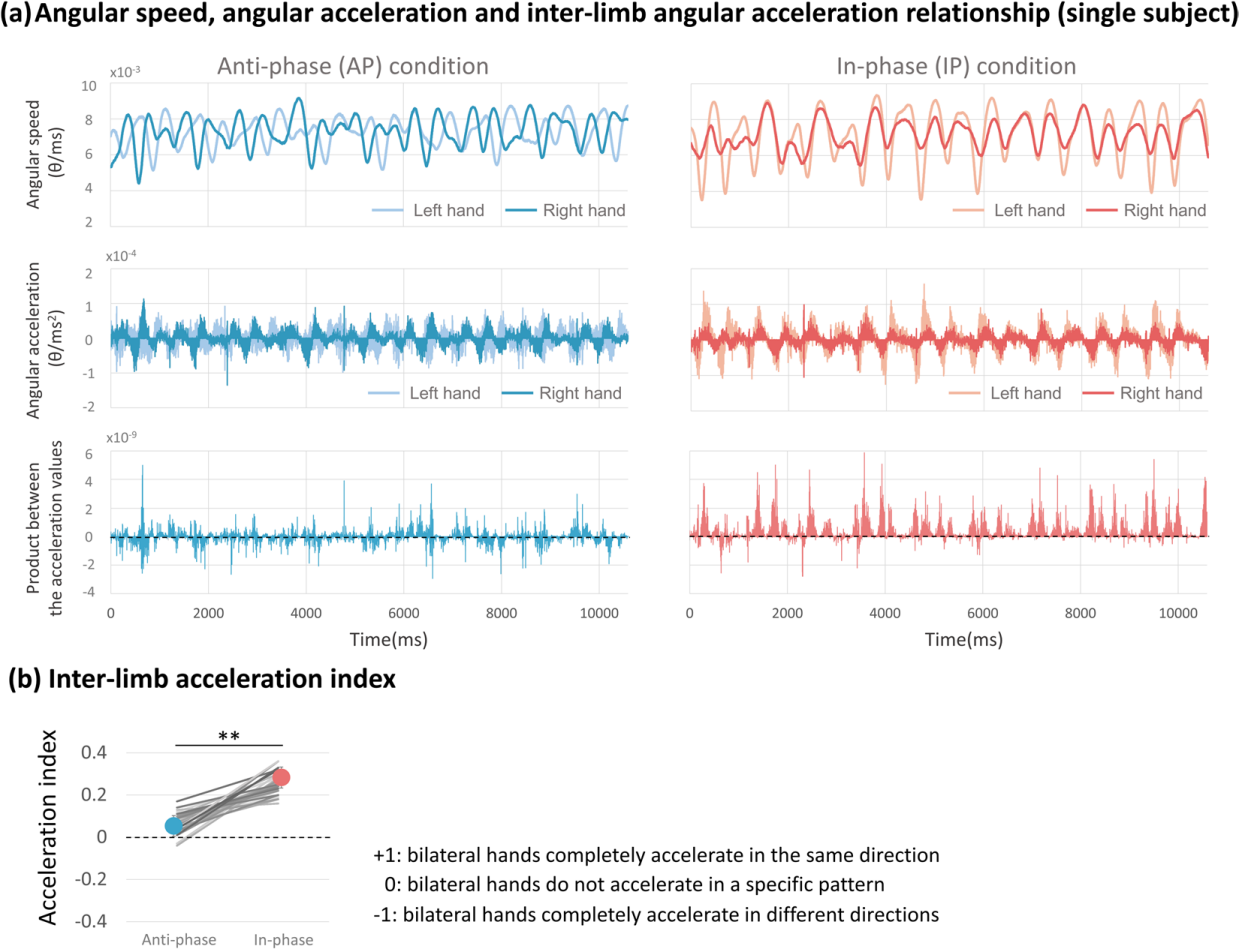
Inter-limb acceleration index. (**a**) An example of angular speed and acceleration fluctuation within the trial (same subject, same trial as Fig. 7). The first row showed the time-series angular speed data from both hands. The speed changes of both hands are more convergent during in-phase movements, while in anti-phase movements the pattern is random. The second row showed the differentiation of the angular speed, i.e., angular acceleration of both hands. The third row showed the product between the time series acceleration values from left and right hands. (**b**) Individual and averaged inter-limb acceleration index during anti-phase movements and in-phase condition. **p < 0.001.

During in-phase movements, bilateral hands have a strong tendency to accelerate with the in-phase relationship, while in anti-phase movements, two hands accelerate and decelerate without a specific relationship (IP: 0.25 ± 0.052, AP: 0.06 ± 0.050; t_(29)_ = −12.557, p < 0.001) (see Fig. 8a for the speed/acceleration profile of an individual subject; Fig. 8b for the group result). This result indicated that in in-phase condition, participants performed the task with a convergent inter-limb hand acceleration relationship; i.e., during in-phase movements, both hands predominantly accelerated and decelerated at the same time, while in anti-phase condition, the inter-limb acceleration profile was random.

## Discussion

In the present study we demonstrate that spatiotemporal movement characteristics are differentially affected by both bilateral coordination patterns. First, in-phase movements are performed with higher spatial consistency than anti-phase movements (i.e., they have lower radial variability). This difference relates to better performance of the non-dominant hand in in-phase movements as compared to anti-phase movements, suggesting that non-dominant hand performance is facilitated during in-phase movements. Second, in-phase movements are performed with greater between-limb synchronization and a more convergent (i.e., in-phase) speed change profile than anti-phase movements. Our results suggest (i) that different control processes govern both coordination modes and (ii) that in-phase movements might have a beneficial effect on kinematic performance in the weaker (non-dominant) limb.

Our results demonstrate kinematic asymmetries between the dominant and non-dominant hands during the control of bilateral movement patterns. Radial variability and peak speed variability were both greater in the non-dominant compared to the dominant hand across all conditions. Interestingly, non-dominant hand performance became more consistent and stable during in-phase movements. This result supports the hypothesis that performance of the non-dominant hand is more prone to be affected by task demand during bilateral movements, while the performance of the dominant hand remains stable^12,13^. Semjen *et al*. (1995) found that with high-frequency circle drawing movements the distortion of the non-dominant hand trajectory was larger during anti-phase movements than during in-phase movements. In addition, there was a higher chance of movement direction reversals happening during anti-phase movements specifically in the non-dominant hand. Our data confirm and extend these results: we found that the non-dominant hand reached better performance during bilateral in-phase movements than anti-phase movements. Our findings are in line with those of Helmuth and Ivry (1996)^27^, who identified a bilateral advantage for timing during finger tapping: lower temporal variability was found during bilateral tapping compared to the unilateral tapping. This observation supports the concept, that in-phase movements have a facilitatory effect on the performance of the “weaker” non-dominant hand, suggesting that a symmetrical movement pattern can improve the temporal stability of the movement. Furthermore, our results demonstrate that this facilitatory effect does not only apply for the temporal, but also for the spatial domain, in a way that the bilateral in-phase advantage can even improve the spatial accuracy. In addition, when estimating the acceleration relationships between hands, our data suggest a temporal advantage for the dominant hand - which is evidenced by the phase lag between hands with the dominant hand in a leading position. Again, this pattern was more prominent in anti-phase movements compared to in-phase movements, which may help confirming that the dominant-hand advantage is more pronounced during anti-phase movements than in-phase movements in the temporal domain^14,16,17^. However, Franz *et al*. (2002) reported that not hand dominance but rather movement direction determines which hand leads^28^. Therefore, the effect of the leading hand might be dependent on the task selection and experimental setup. Taken together, differences in temporal and spatial parameters between the dominant and non-dominant hands decrease during in-phase movements and support the notion that this movement mode represents a basic movement coordination mode with a synergistic control of the hands.

In order to assess synchronization between hands during both bilateral movement modes, we studied the inter-limb phase difference across time series and thereby derived a quantitative index for inter-limb synchronization^26^. This index quantifies the coupling between the performances of both hands. Our data demonstrate a higher inter-limb synchronization during in-phase movements as compared to anti-phase movements, meaning that the phase relationship in in-phase movements was more stable across time. In order to better understand differences in synchronicity between movement conditions, we also analyzed the speed change relationships between hands. During in-phase movements, acceleration of bilateral arms strongly tended to follow an in-phase relationship, while during anti-phase movements, no systematic relationship between hands was found. Taken together, these results provide evidence that during in-phase movements, bilateral movements are highly synchronized and both arms exhibit convergent speed change profiles. This, in turn, suggests that there is strong bilateral coupling during in-phase movements.

Since the cyclic movements in our paradigm were externally paced by an auditory metronome, we additionally asked whether auditory-motor synchronization might have influenced the differential results of both bilateral movement patterns, as suggested previously^29,30^. However, no differences were found for cycle period variability between hands and conditions. Therefore in our paradigm, the variability of auditory-motor synchronization might not affect the outcome of our main kinematic variables that target to differentiate in-phase movements from anti-phase movements.

**Figure 9:**
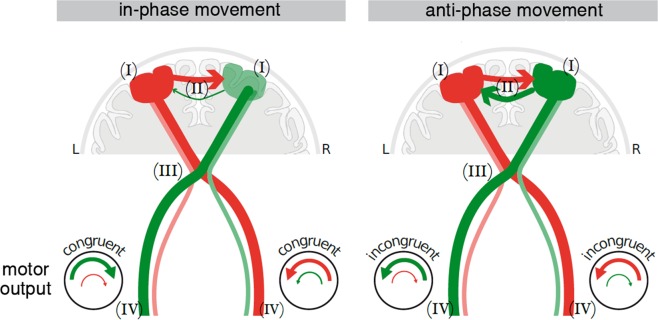
Scheme of interaction levels during in-phase movements and anti-phase movements. (I) in-phase movements are controlled with a left-hemisphere dominance (dark red for left hemisphere, light green for right hemisphere), while anti-phase movements are controlled more independently by both hemispheres (dark red/green for left/right hemisphere); (II) transcallosal pathways mediate left-hemisphere dominance during in-phase movements (thicker arrow from left to right), thereby facilitating the desired congruent movement of the non-dominant cortex; (III) crossed and uncrossed corticofugal fibers modulate the interaction between hands and contribute to the motor output in the periphery (IV): the motor output is elicited by crossed (larger arrow) and uncrossed (smaller arrow) pathways. In in-phase movements, motor outputs from crossed (larger arrow) and uncrossed fibres (smaller arrow) are congruent (synchronous activation of homologous muscles), while in anti-phase movements, motor outputs from crossed and uncrossed fibers are incongruent (synchronous activation of non-homologous muscles).

What might be the mechanisms underlying the differential kinematic traits of both bilateral movement conditions found in our study? From a neuroanatomical and neurophysiological perspective, the structural and functional characteristics of our motor system have made the human body prone to in-phase movements^31,32^. According to previous neuroimaging studies, in-phase movements can be executed with more stable performance, since they are exerted under the preponderant influence of the dominant hemisphere that controls (i) contralateral arm movements by crossing corticospinal pathways and (ii) corticospinal output of the non-dominant motor cortex via interhemispheric projections, thereby eliciting movements of the non-dominant in high synchronicity with the dominant arm (please refer to Fig. 9 for a synopsis)^17,33^. This notion was supported by the findings of a study using transcranial magnetic stimulation (TMS)^34^. During bilateral in-phase movements, TMS pulses applied over one motor cortex interrupts rhythmic movements in both hands, whereas during anti-phase movements, only the contralateral hand is affected. Since our two hands have a strong tendency towards mirror movement pattern, it has been suggested that a non-mirroring transformation network is required to restrict the interaction between hemispheres, especially for the non-dominant hand, to generate the adequate unilateral movement^32,35^. While not directly tested using complementary neurophysiological measures, it is tempting to speculate that similar processes explain our kinematic data of intra-limb variability: here, non-dominant hand performance is improved during in-phase as compared to anti-phase movements, since in this case, no separate control of each hand by a non-mirroring transformation network is needed.

Measurements that capture inter-limb acceleration relationship are particularly informative for pointing to different control processes of one or the other bilateral movement mode: the convergent inter-limb acceleration relationship during in-phase movements can be related to the co-activation of the homologous muscle groups^36–38^, which is a result of not only the transcallosal but also the descending fiber structures^39–42^. It is known that a small proportion of the corticospinal fibers do not cross at the pyramidal decussation, but project to ipsilateral spinal motoneurons^43,44^. Since the descending commands from the motor cortex are sent through both crossed and uncrossed corticospinal fibers, the outputs of crossed and uncrossed descending corticospinal projections to the same limb are congruent (synchronous activation of homologous muscle groups in both pathways) and facilitate the desired movement; therefore, a clear pattern that the two hands consistently accelerate at the same time was observed during an in-phase movements. On the other hand, during anti-phase movements, both crossed and uncrossed pathways to the same limb might result in motor output incongruency/interference (synchronous activation of non-homologous muscles in both pathways). This, in turn, might require more movement speed adjustments that finally leads to a random inter-limb acceleration pattern. The corticospinal structures, therefore, might provide the basis for facilitation or interference between limbs^45,46^.

There are surely other influences that potentially could contribute to our results: First, it was shown previously, that the spontaneous preference for symmetrical movements might be purely perceptual, meaning that the perceptual inputs and cues can crucially influence the movement tendency^47,48^. Second, biomechanical properties of the upper limb during different movement directions could play a role. However, with our experimental design, the contribution of perception has been minimized (participants were only allowed to focus the eyes on the center cross); also, we included two different drawing directions for each condition to reduce the potential bias of the joint property.

The result of a facilitatory effect of the non-dominant hand movements during in-phase movements might be of interest for clinical populations with focal brain lesions and resulting motor deficits. Indeed there is a variety of therapeutic approaches using active bilateral movements to facilitate paretic arm performance, mainly using a mixture of both anti-phase movements and in-phase movements patterns^49^. However, until now, it remains unknown, whether the two different bilateral movement patterns differentially affect performance of the paretic arm. From our results in healthy subjects, we confirmed that the circle drawing task on the KINARM is capable of capturing the difference between the two fundamental coordination patterns. Therefore, this paradigm can be further applied to clinical populations to investigate how neurological diseases affect different coordination patterns. Furthermore, using a robotic device that allows movement/measurement of elbow/shoulder joints and mechanical forces over the joints, opens up the possibility to further extend bilateral coordination research, for example, joint coordination and external perturbation studies on healthy subjects and patients.

In sum, kinematic analyses suggest differential control processes involved in basic bilateral coordination patterns. During in-phase movements, a common neural generator (i.e. the dominant hemisphere) controls movements in both limbs resulting in highly synchronous movements and in-phase acceleration profiles, while anti-phase movements are controlled by both hemispheres more independently leading to less synchronicity and a random between-hand acceleration relationship.

## Supplementary information

Supplementary materials

## Data Availability

Data are available from the corresponding authors upon reasonable request.
