# Supplementary material for: Kinematic profiles suggest differential control processes involved in bilateral in-phase and anti-phase movements
Source: Sci Rep. 2019 Mar 1;9:3273. doi: 10.1038/s41598-019-40295-1 (PMC6397147; doi:10.1038/s41598-019-40295-1)
Supplement: Supplementary file 1 — Supplementary materials [file 41598_2019_40295_MOESM1_ESM.pdf]

Supplementary Information

Kinematic profiles suggest differential control processes involved in bilateral in-phase and anti-phase movements

Pei-Cheng Shih[1], Christopher J. Steele[1,2], Vadim Nikulin[1], Arno Villringer[1,3], Bernhard Sehm[1,3,*]

[1] Department of Neurology, Max Planck Institute for Human Cognitive and Brain Sciences, Leipzig, Germany

[2] Department of Psychology, Concordia University, Montreal, Quebec, Canada

[3] Department of Cognitive Neurology, University of Leipzig, Leipzig, Germany

[*] sehm@cbs.mpg.de

Supplementary material 1. Power estimation

We conducted a power analysis to examine how effect size relates to the minimum sample size required for a given power and an alpha of 0.05. Most of the previous studies on bilateral coordination had a sample size between 8 to 16 participants and an effect size between 0.65 to 0.85 in the main result. However, this would provide a meager statistical power regardless if effect size is high (>0.8) or moderate (0.5 - 0.8). On the other hand, a sample size at 30 results in a high power. Therefore, our sample size allowed us to detect differences between movement conditions within a subject that may have been previously missed.

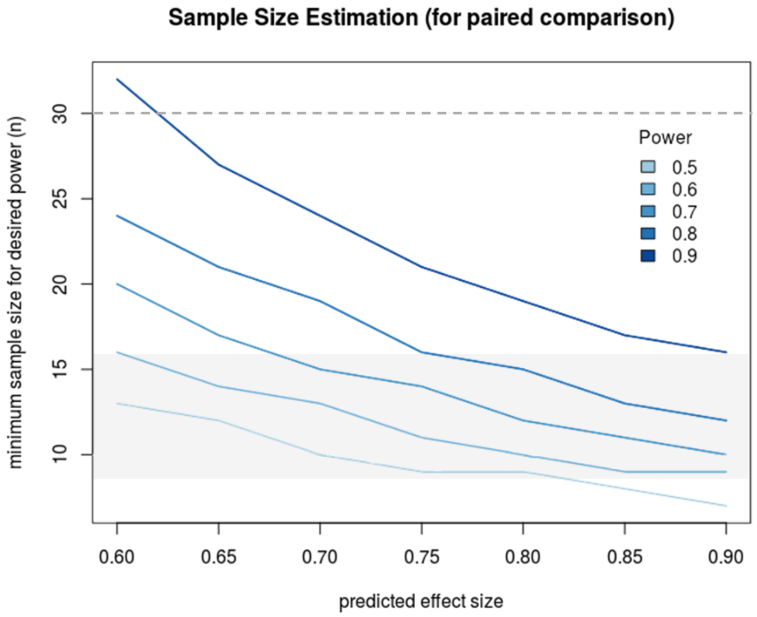

Supplementary material 2. Participants' performance across blocks of task execution.

<u>Radial variability</u>

A one-way repeated-measure ANOVA was used to determine the effect of BLOCK on each condition/hand. As shown in the figure below, no learning effects were found in both anti-phase and in-phase conditions, and in left and right hand performance.

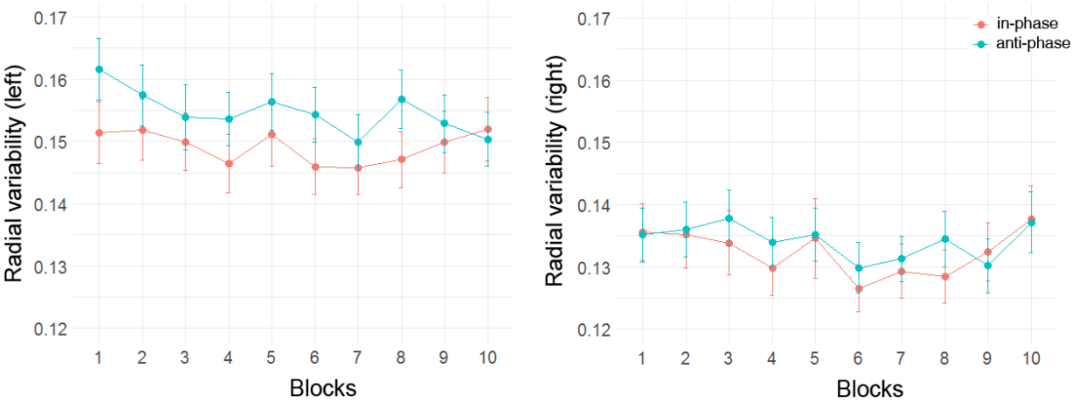

Condition	Statistical result
Anti-phase (left hand)	$F_{(9,261)} = 1.001$, $p = 0.439$, $\eta^2 = 0.033$
Anti-phase (right hand)	$F_{(9,261)} = 1.604$, $p = 0.114$, $\eta^2 = 0.052$
In-phase (left hand)	$F_{(9,261)} = 1.540$, $p = 0.161$, $\eta^2 = 0.050$
In-phase (right hand)	$F_{(9,261)} = 1.868$, $p = 0.090$, $\eta^2 = 0.061$

<u>Synchronization index</u>

We performed one-way repeated-measure ANOVA was used to determine the effect of BLOCK on anti-phase and in-phase movements respectively. No learning effects were found across the blocks in both anti-phase and in-phase conditions as shown in the figure below.

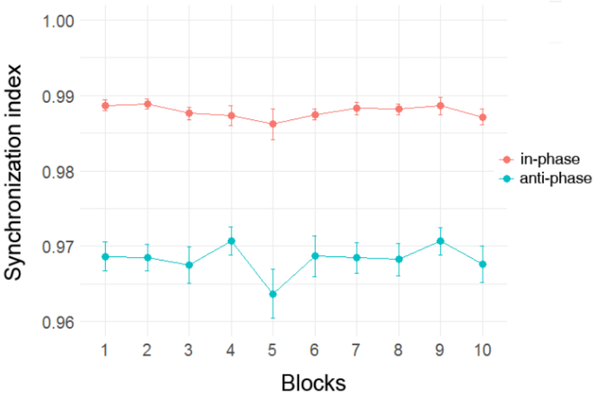

Condition	Statistical result
Anti-phase	$F_{(9,261)} = 1.604$, $p = 0.114$, η2 =0.052
In-phase	$F_{(9,261)} = 1.047$, $p = 0.403$, η2 =0.035

Supplementary material 3. Comparison of clockwise and counterclockwise movement directions.

Radial variability

Unilateral condition	Clockwise	Counterclockwise	Paired-t test
Left hand	0.166±0.033	0.144±0.024	t=6.1201, p<0.001
Right hand	0.134±0.026	0.138±0.032	t=-2.4314, p=0.0263

Anti-phase condition	Bilateral clockwise	Bilateral counterclockwise	Paired-t test
Left hand	0.161±0.032	0.155±0.021	t=2.4938, p=0.0186
Right hand	0.137±0.025	0.137±0.031	t= 0.1767, p=0.8617

In-phase condition	left clockwise / right counterclockwise	left counterclockwise / right clockwise	Paired-t test
Left hand	0.158±0.033	0.142±0.022	t=5.3041, p<0.001
Right hand	0.136±0.032	0.134±0.028	t=1.0794, p=0.2893

Synchronization index

Anti-phase condition	Bilateral clockwise	Bilateral counterclockwise	Paired-t test
Synchronization index	0.965±0.001	0.963±0.001	t= -1.6214, p=0.1158

In-phase condition	left clockwise / right counterclockwise	left counterclockwise / right clockwise	Paired-t test
Synchronization index	0.984±0.001	0.985±0.001	t= -1.1266, p=0.2692

Supplementary material 4. Centroid offset during the in-phase and anti-phase phase conditions

Centroid offset	Left hand	Right hand
Anti-phase	0.789±0.1270	0.6760±0.1228
In-phase	0.7273±0.1305	0.6591±0.1190

2x2 repeated-measured ANOVA

	F-value	P-value
CONDITION*SIDE	4.4865	<0.0360
CONDITION	13.904	0.0003
SIDE	74.225	<0.001

Supplementary material 5. Supplementary Material 4. Comparison of phase synchronization index and standard deviation of the relative phase difference

Phase synchronization

Cond	Mean ± SD	t	P
In-phase	0.98±.001	8.276	<0.001
Anti-phase	0.96±.001		

SD of the relative phase difference

Cond	Mean ± SD	t	P
In-phase	9.69±6.03	5.205	<0.001
Anti-phase	14.33±2.93		
